# Wegener’s granulomatosis with orbital involvement: case report and literature review

**DOI:** 10.22336/rjo.2021.19

**Published:** 2021

**Authors:** Andrada-Elena Mirescu, Ioana Teodora Tofolean, Mihaela Florica Milicescu, Irina-Elena Cristescu, Andrei Teodor Iacob, Florian Baltă

**Affiliations:** * Emergency Eye Hospital, Bucharest, Romania; ** “Carol Davila” University of Medicine and Pharmacy, Bucharest, Romania; *** “Retina” Clinic, Bucharest, Romania; **** Rheumatology Department, “Dr. Ioan Cantacuzino” Clinical Hospital, Bucharest, Romania

**Keywords:** granulomatosis with polyangiitis, ANCA associated vasculitis, orbital granuloma, anti-CD20 monoclonal antibodies

## Abstract

**Objective:** To describe the chronology and the extent of orbital involvement in a case of granulomatosis with polyangiitis.

**Methods:** Descriptive case report and literature review.

**Results:** A 45-year-old patient, formerly diagnosed with granulomatosis with polyangiitis due to otorhinolaryngologic manifestations, pulmonary lesions, renal impairment, left knee arthritis and high blood levels of antineutrophil cytoplasmic antibodies, addressed the Ophthalmology Department in November 2020, having the following complaints: left eye mild retro-orbital discomfort, proptosis and epiphora.

On examination, Snellen’s best corrected visual acuity was 6/ 6 in both eyes. The anterior segment of the left eye displayed significant changes: proptosis, upper lid swelling, ptosis, slightly decreased ocular motility, mild conjunctival hyperemia and chemosis, thinning of sclera in the upper quadrant and mild posterior subcapsular cataract. Left eye funduscopy revealed a slightly elevated optic disc, with indistinct margins in the nasal quadrant. Spectral-domain optical coherence tomography (OCT) of the optic nerve confirmed the clinical findings, illustrating an increase in the retinal nerve fiber layers thickness in the nasal quadrant, with no corresponding visual field defect. The orbit magnetic resonance imaging (MRI) unveiled an intraconal mass surrounding the optic nerve on its entire intra-orbital path, confirming the diagnosis of left orbital granuloma.

**Conclusion:** Considering the relapsing disease and the orbital involvement, the patient is currently a candidate for rituximab, a monoclonal antibody against CD20.

**Abbreviations:** AAV = ANCA associated vasculitides, ANCA = antineutrophil cytoplasmic antibody, AOM = acute otitis media, BCVA = best corrected visual acuity, CRP = C-reactive protein, CT = computerized tomography, EGPA = eosinophilic granulomatosis with polyangiitis, ENT = otorhinolaryngology/ ear-nose-throat, ESR = erythrocyte sedimentation rate, GPA = granulomatosis with polyangiitis, LE = left eye, MPA = microscopic polyangiitis, MRI = magnetic resonance imaging, OCT = optical coherence tomography, RE = right eye, RNFL = retinal nerve fiber layers, TNF = tumor necrosis factor, WG = Wegener’s granulomatosis

## Introduction

Granulomatosis with polyangiitis (GPA), more commonly referred to as Wegener’s granulomatosis (WG), is a necrotizing vasculitis that affects small and medium-sized vessels. According to the modern nomenclature of systemic vasculitides introduced in 2012 at the Chapel Hill Consensus Conference, the condition belongs to the group of antineutrophil cytoplasmic antibody (ANCA) associated vasculitides (AAV), a broad spectrum of disorders that encompasses, in addition to GPA, microscopic polyangiitis (MPA) and eosinophilic granulomatosis with polyangiitis (EGPA) (Churg-Strauss syndrome) [**1**]. 

The worldwide incidence of GPA ranges from 10 to 20 cases per one million inhabitants, depending on the geographic area. GPA can affect all racial groups, but appears predominantly in Caucasians, showing no sex predilection [**2**].

The etiopathogenesis of GPA is not yet completely understood, presuming an altered immune response to specific environmental factors in genetically predisposed individuals. Serum anti-neutrophilic cytoplasmic antibodies (ANCA), found in 80-90% of the patients with active systemic condition, represent the hallmark of the disease [**3**]. Although c-ANCA (with proteinase 3 specificity) mostly appear in Wegener’s granulomatosis (80-90% of ANCA-positive GPA patients), and p-ANCA (directed against myeloperoxidase) are mainly found in microscopic polyangiitis, 10-20% of ANCA-positive GPA patients have increased p-ANCA levels. The antibody titers in the serum are usually high at presentation, decrease with treatment and increase again during the progression of the disease, announcing relapses [**4**].

The granulomas in GPA embody giant cells, plasma cells, dendritic cells, and lymphocytes, causing partial or total occlusion of blood vessels. Activated cells might invade the surrounding tissue, causing necrosis and thus permanent damage [**3**,**5**].

Wegener granulomatosis usually presents with non-specific general symptomatology (fever, malaise, weight loss, arthralgia, myalgia) and commonly involves the upper respiratory tract/ ear-nose-throat (otitis media, mastoiditis, hearing loss, sinusitis, rhinitis), the lower respiratory tract (lung nodules, alveolar hemorrhage) and the urinary system (glomerulonephritis) [**6**]. Other affected organs/ systems are: the eye (more than half of the patients), the skin (50-60%: lower extremities purpura, cutaneous nodules, ulcers, papules, vesicles, subcutaneous nodules), the nervous system (30-40%: peripheral neuropathy, mononeuritis multiplex, cranial neuropathies, pachymeningitis, seizures, cerebritis), the musculoskeletal system (70%: arthralgia, myalgia, arthritis) and the heart (rare, valvular lesions, pericarditis, coronary arteritis) [**6**].

## Methods

The present report describes the case of a forty-five-year-old male previously diagnosed with granulomatosis with polyangiitis, due to upper and lower respiratory tract, kidney, and musculoskeletal involvement. Ocular symptomatology appeared at some point during the disease. The current paper follows the chronology and the extent of the ocular features in the wider context of a multisystem disorder. 

## Results

 In June 2017, a 42-year-old male known with supraventricular tachycardia treated with verapamil (120 mg/ day), experienced an episode of severe right otalgia and decreased hearing. After ENT admission, the diagnosis of acute otitis media (AOM) was confirmed and the patient underwent a right ear myringotomy, further treated with analgesics, antibiotics, and anti-inflammatory drugs. Because acute otitis media occurs much more commonly in children than in adults, supplementary investigations were made, revealing high blood levels of inflammation markers (CRP, ESR, fibrinogen, leukocytes, granulocytes), mixed hearing loss, mild mastoiditis, chronic atrophic rhinitis, nasal ulcers, and nasal septum deviation. 

Although the right ear pain and discharge improved in a couple of days, the patient’s decreased hearing and inflammation markers were not resolving and the general condition was deteriorating (headaches, diffuse arthralgia, and myalgia). Under the given circumstances, an autoimmune disorder was suspected and the patient was referred to the Rheumatology Department.

**Fig. 1:**
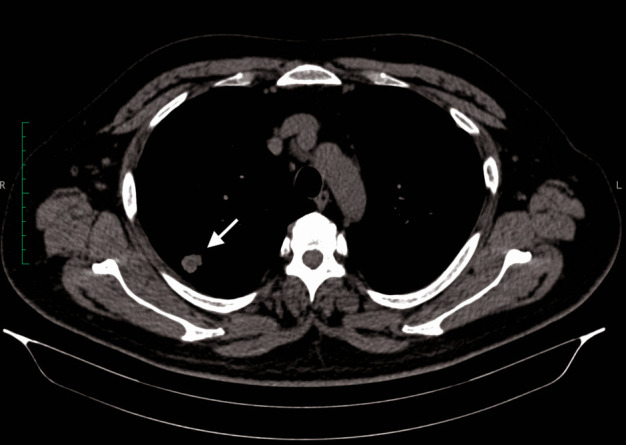
Lung CT-scan: solitary pulmonary nodule situated in the posterior segment of the right lung’s superior lobe (white arrow)

After an extensive work-up, considering the otorhinolaryngologic manifestations (acute otitis media with mastoid involvement, nasal ulcers, chronic atrophic rhinitis, mixed hearing loss), the pulmonary lesions (solitary pulmonary nodule situated in the superior lobe of the right lung, **Fig. 1**), the renal impairment (microalbuminuria), the left knee arthritis, the high blood levels of inflammation markers and antineutrophil cytoplasmic antibodies (c-ANCA), the diagnostic criteria for active systemic granulomatosis with polyangiitis were met.

The induction therapy of choice was an intermittent pulse therapy (6-months regimen, monthly administration) with intravenous methylprednisolone (total dose: 1.25-1.5 g) and intravenous cyclophosphamide (total dose: 1 g) plus oral prednisolone (starting dose 40 mg/ day, tapering 2.5 mg every two weeks). Six months later, considering the patient’s favorable evolution (clinical condition, blood levels of inflammation markers, c-ANCA titer, pulmonary CT-scan), the treatment responsible for remission induction was switched to maintenance therapy (oral prednisolone with constant tapering rate and azathioprine 100 mg/ day).

Only one month later, in January 2018 (prednisolone 15 mg/ day, azathioprine 100 mg/ day), the first ocular symptom appeared: binocular diplopia. It resolved one week later, after increasing the doses of corticosteroids (methylprednisolone 64 mg/ day, tapering 8 mg every week) and immunosuppressive medication (azathioprine 150 mg/ day).

In November 2020, the 45-year-old patient (at that time) presented in the Ophthalmology Department with the following complaints: left eye mild retro-orbital discomfort, proptosis and epiphora. The symptomatology initially appeared approximately one year before, improved after self-conducted corticosteroids’ dose increase and recurred while tapering. 

**Fig. 2:**
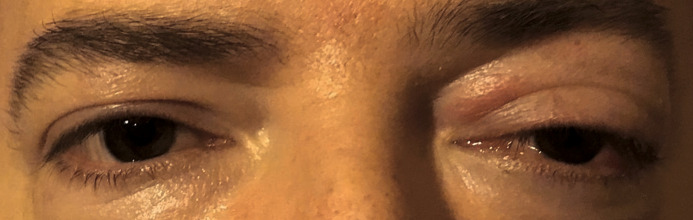
Right eye: normal appearance; Left eye: proptosis, upper lid swelling, ptosis, mild conjunctival hyperemia and chemosis

On examination, Snellen’s best corrected visual acuity (BCVA) was 6/ 6. Intraocular pressure values were within normal limits in both eyes. The anterior segment proved to be normal in the right eye (RE), but the left eye (LE) displayed significant changes: proptosis, upper lid swelling, ptosis, slightly decreased ocular motility, mild conjunctival hyperemia and chemosis, thinning of sclera in the upper quadrant and mild posterior subcapsular cataract (**Fig. 2**). RE fundus exam was within normal limits, but the LE funduscopy revealed a slightly elevated optic disc, with indistinct margins in the nasal quadrant. 

**Fig. 3:**
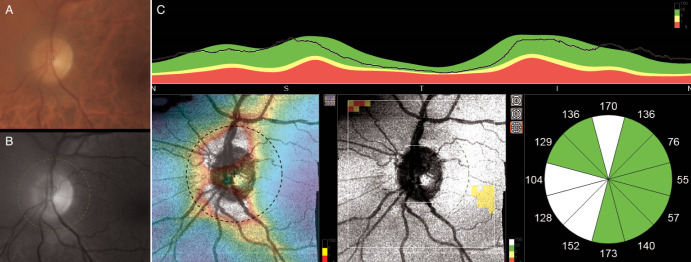
**A.** Color and **B.** Red-free fundus photo of the left eye showing a slightly elevated optic disc, with indistinct margins in the nasal quadrant. **C.** Spectral-domain optical coherence tomography (OCT) of the optic nerve illustrating an increase in the retinal nerve fiber layers thickness in the nasal quadrant of the left eye

**Fig. 4:**
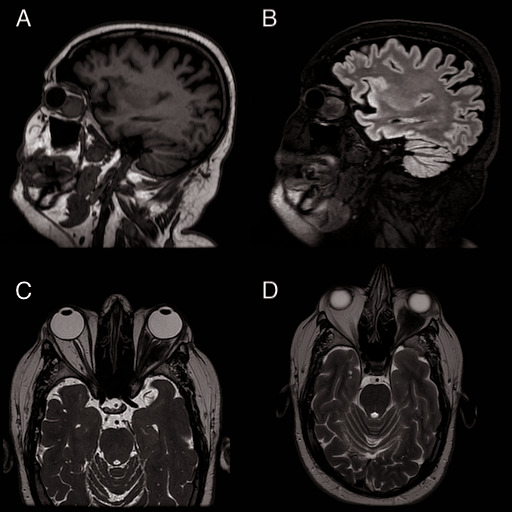
Magnetic resonance imaging (MRI) of the orbit and brain without contrast agent revealed an intraconal mass, whose maximum dimensions are 24.7/ 25.3/ 27.3 mm (in craniocaudal, anteroposterior, and transverse planes), that surrounds the optic nerve on its entire intra-orbital path, with obliteration of adjacent fat planes in the left orbit. **A.** High resolution three-dimensional T1-weighted gradient image (BRAVO), sagittal plane. **B.** T2-weighed volume sequence (CUBE) with fluid-attenuated inversion recovery (FLAIR) contrast, sagittal plane **C.** T2-weighed volume sequence (CUBE), axial plane **D.** T2-weighed fast recovery fast spin echo sequence (FRFSE), sagittal plane

Spectral-domain optical coherence tomography (OCT) of the macular region confirmed the absence of any macular lesions, while the optic nerve scans attested the clinical findings, thus illustrating an increase in the retinal nerve fiber layers (RNFL) thickness in the nasal quadrant of the left eye (**Fig. 3**). Visual fields showed no significant changes. Orbit magnetic resonance imaging (MRI) unveiled an intraconal mass surrounding the optic nerve on its entire intra-orbital path, with obliteration of adjacent fat planes in the left orbit (**Fig. 4**). The diagnosis of left orbital granuloma was made, based on the corroboration of medical history, clinical findings, c-ANCA titers and radiological data. 

## Discussion

Orbital involvement in GPA is rare, found in about 15% of the cases, representing about 45% of ocular manifestations of the disease and being frequently associated with the limited, benign form of the illness [**7**,**8**].

According to a large, multi-center report, orbital involvement in GPA encompasses: lid erythema, proptosis, or orbital mass (69%), obstruction of the nasolacrimal duct (52%), lacrimal sac mucocele (47%), extraocular muscle involvement or diplopia (52%), bony erosion, orbital socket contracture, enophthalmos and compressive optic neuropathy. 80% of the compressive neuropathy cases are due to the presence of an orbital granuloma and 14% of them are related to bilateral lacrimal gland masses [**8**]. 

The current report outlined a new case of non-life-threatening Wegener’s granulomatosis with orbital involvement, firstly documented as an isolated episode of diplopia that occurred one month after the end of induction therapy, during the maintenance phase. Two silent years later, while still on maintenance therapy, orbital symptomatology returned. The patient experienced insidious onset and slowly progressive left eye proptosis with subsequent ocular manifestations (upper lid swelling, ptosis, slightly decreased ocular motility, mild conjunctival hyperemia and chemosis), due to a gradually developing orbital granuloma. Luckily, no significant left optic nerve compression and no associated visual field defects or decreased vision were present. Interestingly, orbital granuloma development was not associated with significant blood levels of inflammation markers and antineutrophil cytoplasmic antibodies increase nor with any other disease-related symptomatology.

The main goals of the treatment in GPA are remission induction, followed by maintenance of remission, by using immunosuppressant drugs. Oral corticosteroids alone failed to induce remission [**9**]. Drugs with proved effectiveness in induction and/ or maintenance of remission are: cyclophosphamide, methotrexate, rituximab, azathioprine, trimethoprim-sulfamethoxazole, mycophenolate mofetil, 15-deoxyspergualin, cyclosporine, and intravenous immunoglobulin [**10**-**15**]. Tumor necrosis factor (TNF)-α antagonists were also assessed. Infliximab was effective only in small clinical trials, while etanercept, another member of the family, showed no significant advantage over placebo in remission maintenance [**12**].

The cyclophosphamide and high-dose glucocorticoids combination is the standard of care for remission-induction in GPA patients, usually lasting for 3 to 6 months. A randomized controlled trial investigated the efficacy of rituximab (a chimeric monoclonal antibody against CD20) in ANCA-associated vasculitis, suggesting that treatment with rituximab and glucocorticoids is not inferior to the standard regimen in patients with severe ANCA-associated vasculitis of recent onset and may be superior in relapsing disease [**16**]. Another frequently chosen option is methotrexate – glucocorticoids combination.

In order to avoid relapses after remission achievement, maintenance therapy is initiated, usually for 12 to 36 months. Methotrexate, azathioprine, and rituximab have proven their potential, the rate of sustained remission for ANCA-associated vasculitis patients following rituximab-based maintenance regimens remaining superior over azathioprine-based regimens after 60 months of observation, with better overall survival [**17**].

Due to the relapsing disease and the orbital involvement [**18**,**19**], the patient is currently a candidate for rituximab, which offers an alternative for the induction therapy in patients with Wegener’s granulomatosis, having superior effectiveness when used as a maintenance immunosuppressive agent as well. 

## Conclusion

Considering the important side effects of rituximab, the COVID-19 pandemic, the patient’s level of (social) activity, the single-organ involvement, the excellent response and good tolerance to the initial induction therapy and the national consensus on the use of rituximab in GPA patients, the doctor-patient agreement led to another round of cyclophosphamide-glucocorticoids regimen for remission induction. In case of failure of the current approach, the patient will be switched to anti-CD20 therapy. 

Although associated with significant mortality and morbidity, introduction of immunosuppressive agents and biologics have significantly improved the long-term survival rate and the quality of life for GPA patients. 

**Authors’ contribution**

All authors have equal contribution.

**Conflict of Interest**

The authors declare no conflict of interest.

**Informed Consent and Human and Animal Rights statements**

Informed consent has been obtained from all individuals included in this study.

**Authorization for the use of human subjects**

Ethical approval: The research related to human use complies with all the relevant national regulations, institutional policies, is in accordance with the tenets of the Helsinki Declaration, and has been approved by the Ethics Committee of “Carol Davila” University of Medicine and Pharmacy, Bucharest, Romania.

**Acknowledgements**

None.

**Sources of Funding**

None.

**Disclosures**

None. 
